# In-Situ Observation of Membrane Protein Folding during Cell-Free Expression

**DOI:** 10.1371/journal.pone.0151051

**Published:** 2016-03-15

**Authors:** Axel Baumann, Silke Kerruth, Jörg Fitter, Georg Büldt, Joachim Heberle, Ramona Schlesinger, Kenichi Ataka

**Affiliations:** 1 Forschungszentrum Jülich, Institute of Complex Systems, Molecular Biophysics (ICS-5), 52425 Jülich, Germany; 2 Freie Universität Berlin, Department of Physics, Experimental Molecular Biophysics, Arnimallee 14, 14195 Berlin, Germany; 3 Moscow Institute of Physics and Technology, Laboratory for Advanced Studies of Membrane Proteins, 141700 Dolgoprudniy, Russia; 4 Freie Universität Berlin, Department of Physics, Genetic Biophysics, Arnimallee 14, 14195 Berlin, Germany; 5 Physikalisches Institut (IA), AG Biophysik, RWTH Aachen, Sommerfeldstrasse 14, 52074 Aachen, Germany; University of Bern, SWITZERLAND

## Abstract

Proper insertion, folding and assembly of functional proteins in biological membranes are key processes to warrant activity of a living cell. Here, we present a novel approach to trace folding and insertion of a nascent membrane protein leaving the ribosome and penetrating the bilayer. Surface Enhanced IR Absorption Spectroscopy selectively monitored insertion and folding of membrane proteins during cell-free expression in a label-free and non-invasive manner. Protein synthesis was performed in an optical cell containing a prism covered with a thin gold film with nanodiscs on top, providing an artificial lipid bilayer for folding. In a pilot experiment, the folding pathway of bacteriorhodopsin via various secondary and tertiary structures was visualized. Thus, a methodology is established with which the folding reaction of other more complex membrane proteins can be observed during protein biosynthesis (*in situ* and *in operando*) at molecular resolution.

## Introduction

The fundamental importance of protein folding as key factor regarding the structure-function relationship of proteins is widely recognized. Study of such highly complex self-organizing processes, that assemble proteins starting from disordered polypeptide chains yielding structures of elaborated biological machineries, are crucial but also challenging. Over the past decades, considerable progress has been made in understanding the folding of water-soluble proteins [1]. This situation contrasts to the knowledge and understanding of the folding pathways of membrane proteins [2–5]. Seminal work on membrane protein folding was performed by Khorana and coworkers in the early 1980s showing that bacteriorhodopsin (bR), a seven-transmembrane (TM) α-helical protein acting as a proton pump in the archaea *Halobacterium salinarum*, can spontaneously recover its native three dimensional structure in detergent after denaturation by organic solvents [6, 7]. This experimental concept of denaturation/renaturation has been extended to other α-helical membrane proteins [2, 8, 9] as well as a few β-barrel membrane proteins [10]. Still, bR is frequently used in studies on membrane protein folding because it is a relatively small polytopic membrane protein with the endogenous retinal chromophore as an indicator for the folded state [11–17]. Development of advanced biophysical methods such as atomic force microscopy enable to observe a folding event at single-protein level [18].

Despite the thermodynamical insight into the folding process gained by renaturation studies, a fundamental question remains: “Do these models actually reflect the folding mechanism of membrane proteins *in vivo*?” It is known that commonly used denaturation chemicals such as SDS, guanidine hydrochloride (GdnHCl), or urea can lead to incomplete unfolding of α-helical membrane proteins [7, 14]. Furthermore, complete unfolding of membrane protein often leads to irreversible denaturation [19]. In contrast to this behavior, the folding process *in vivo* advances from a nascent polypeptide chain leaving the ribosomal tunnel, to the final functional state. Another major obstacle is the lipid bilayer as the folding milieu, which does not provide a simple (homogeneous) hydrophobic environment but a steep gradient in hydrophilicity towards the head groups of the lipids [3].

**Fig 1:**
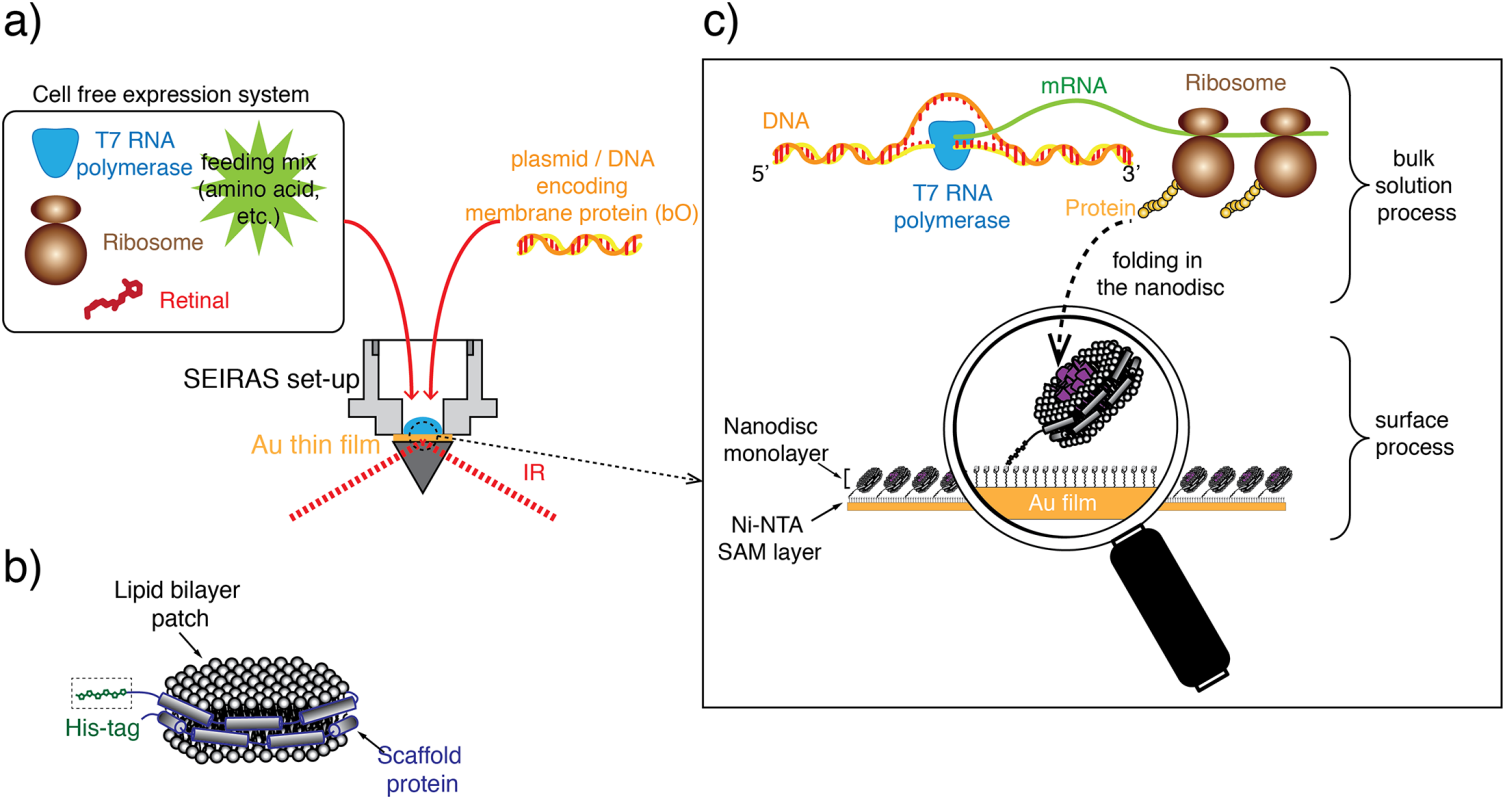
Outline of the experimental concept. (a) SEIRAS optical cell with a droplet of the cell-free expression system to produce bacteriorhodopsin (bR) after addition of the encoding DNA. (b) Nanodisc as biomimetic membrane comprising a phospholipid bilayer (DMPC) wrapped by two amphiphilic membrane scaffold proteins. Each of the latter carry an N-terminal His-tag to bind the nanodiscs to a Ni-NTA modified surface. (c) *In vitro* transcription and translation system synthesizing the polypeptides, which insert into the surface-tethered monolayer of nanodiscs. As the surface enhancement decays exponentially with the distance with an effective length of about 10 nm, processes are exclusively probed by SEIRAS that occur at and within the nanodisc monolayer.

It is obvious that the folding process should ideally be studied under native conditions. However, the folding mechanism is experimentally difficult to address in the complex context of a living cell. Recent developments of cell-free protein expression systems circumvent these constraints. These cell-free systems comprise the essential components for transcription and translation [20–22] of membrane proteins [23–25]. A proper folding milieu for integral membrane proteins is provided by nanodiscs, which are discoidal lipid bilayers wrapped by two amphiphilic membrane scaffold proteins in a belt-like configuration (Fig 1b). Nanodiscs represent the unique advantage of investigating one folding experiment in two different approaches synchronously, in batch and on the surface. This allows the quantitative and qualitative control of the cell-free protein expression for every experiment on a level, which cannot be provided by classical bilayer models like liposomes or lipid monolayers. Elucidating the folding mechanism of membrane proteins requires a method that owes not only molecular sensitivity to resolve the structural changes of the nascent polypeptide chain but also temporal resolution to trace the folding dynamics. Within these boundary conditions, IR spectroscopy has a proven record for molecular studies where structural changes have been monitored at utmost temporal resolution and spatial sensitivity. Furthermore, exploiting plasmonic effects adds selectivity to IR spectroscopy. Here, Surface-Enhanced Infrared Absorption Spectroscopy (SEIRAS) [26–30] exclusively monitors processes that take place in the biomimetic membrane because the enhancement exerted by a rough gold surface is limited to only about 10 nm from the plasmonic gold layer [31] to which the membrane is tethered to (solid-supported membrane). This length scale competes with the typical thickness of 5 nm of a biological membrane. In the present work, we combine a cell-free expression system to monitor membrane protein folding into nanodiscs during transcription/translation with SEIRAS (Fig 1a). In this approach, nanodiscs (Fig 1b and 1c) are immobilized via a His-tag onto a gold surface, which was modified by a self-assembled monolayer (SAM) of nickel chelating nitrilotriacetic acid (Ni-NTA) [32, 33]. The apo-form of the target membrane protein bR, bacterioopsin (bO), is expressed by a cell-free expression system in the bulk solution atop the nanodisc monolayer. As the nascent polypeptide is formed during the transcription/translation process, it diffuses to contact the membrane surface and inserts into the nanodisc lipid bilayer (Fig 1c). The assembly of functional bR requires incorporation of the cofactor retinal into bO. Due to the near field effect of the SEIRAS, the insertion and folding of the nascent polypeptide chain into immobilized nanodiscs are exclusively observed, while signals from processes that occur in the bulk are essentially invisible.

## Results

A cell-free expression system was used to study the insertion and folding of the membrane protein bR into nanodiscs that were bound to a gold surface via a Ni-NTA linker. Expression of the membrane protein was initiated by adding the plasmid-DNA that encodes bO. The cofactor retinal was also present. Shortly after transcription/translation started, the nascent polypeptide contacted the surface-tethered nanodiscs and inserted into the lipid double layer. These membrane-associated processes were traced *in situ* by SEIRAS.

**Fig 2:**
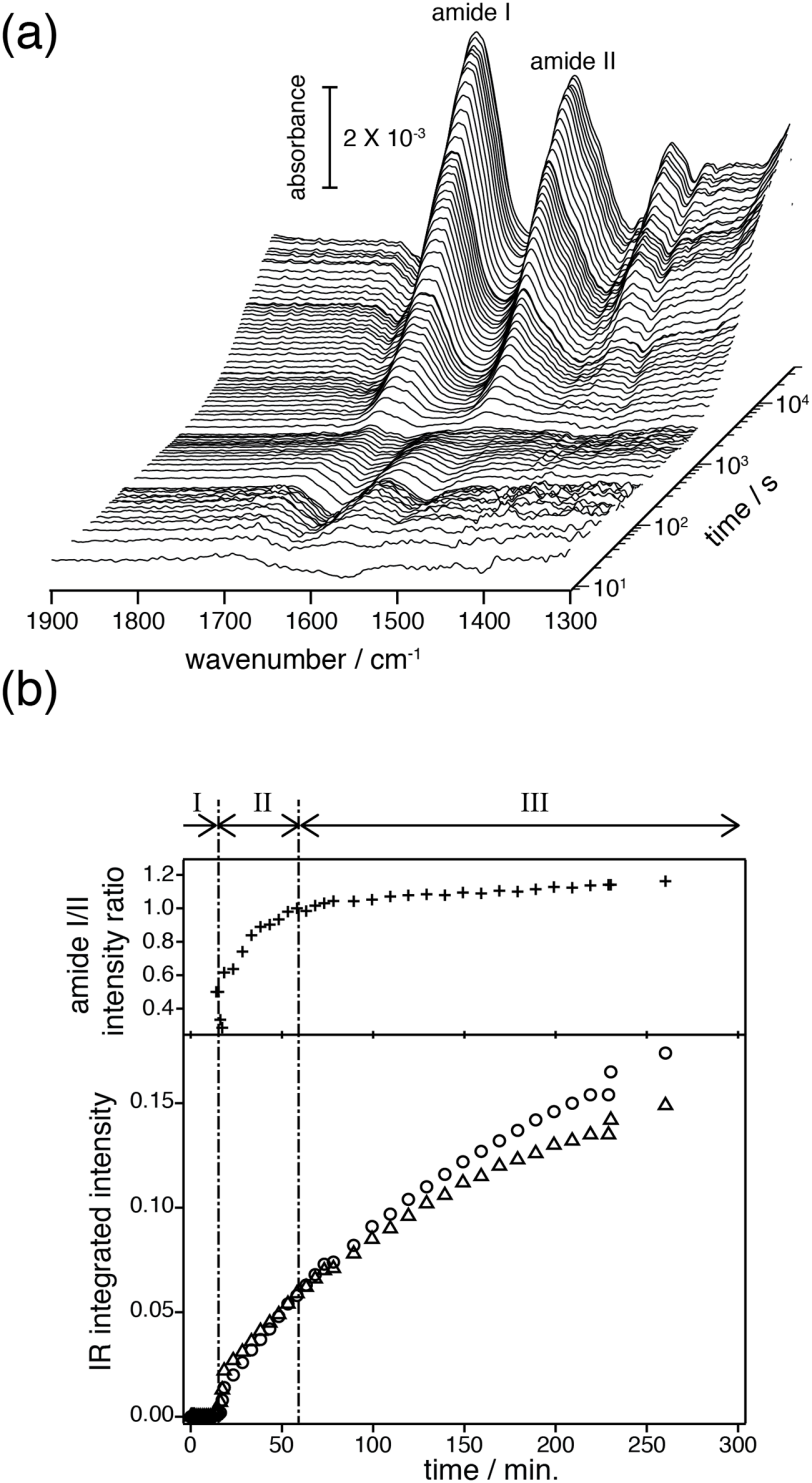
Overview on the time-resolved SEIRAS spectra and their kinetics. (a) Membrane protein folding into the solid-supported nanodiscs observed as spectral evolution after triggering the transcriptional/translational process. (b) Integrated intensities of amide I (○) and II (△) bands (lower) and the intensity ratio of amide I / amide II (upper) are plotted against the reaction time. Roman numbers above the upper part of the plot refer to the I) Pre-conditioning period prior to protein insertion, II) Membrane insertion of the polypeptide chain and formation of secondary structure, and III) Formation of tertiary structure.

Fig 2a represents a series of SEIRA spectra recorded after gene transcription and translation were initiated. The time trace is shown on a logarithmic scale to cover the broad time scale from 10 seconds to 5 hours. After an initial lag phase, two prominent peaks appeared at around 1660 and 1550 cm^-1^ that increased in intensities as time progresses. These bands were assigned to the amide I mode (predominantly C = O stretching vibration of the polypeptide) and amide II mode (coupled mode of the C = N stretching and the N-H in-plane bending vibrations) of the peptide bonds [34]. The plot of the integrated area of the amide I and amide II bands versus time (Fig 2b lower part) shows that protein insertion into the lipid layer was observed only after about 10 minutes. After insertion started, the intensities of the amide bands gradually increased and start to saturate after 4 to 5 hours. During this time, the recorded SEIRA spectra changed significantly. Based on the analysis of the amide I bands, we were able to resolve the insertion and folding of bO.

### Pre-conditioning period prior to protein insertion (0–10 min)

**Fig 3:**
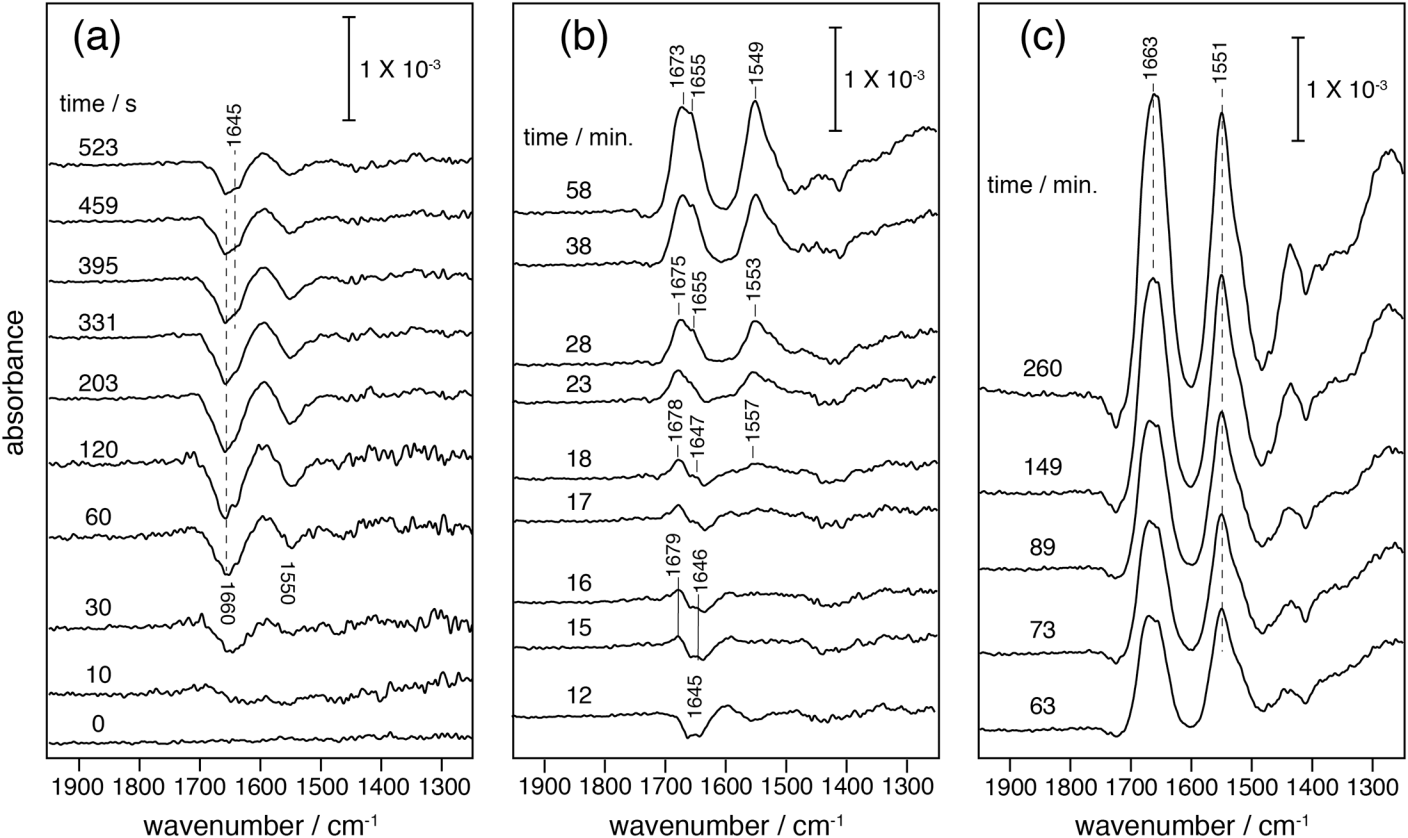
Time-resolved SEIRA spectra recorded after triggering transcription/translation (time = 0 seconds) by adding the plasmid coding for bO. In each spectrum, positive peaks represent species adsorbed (inserted) to (in) the surface tethered nanodiscs, while negative peaks represent species leaving from the nanodisc surface. (a) Pre-conditioning period (ca. 0 to 10 minutes). (b) ca. 10 to 60 minutes: insertion and folding of newly synthesized bR were visible by appearance of positive peaks of amide I (1645–1655, 1679–1673 cm^-1^) and amide II (1557–1549 cm^-1^). (c) 60 minutes to 5 hours: the frequencies of peak maxima of amide I / II of the newly synthesized bR remained constant at 1663 and 1551 cm^-1^, respectively. Note, the width of the amide I bands narrowed with increasing intensity. Background signals from the Ni-NTA SAM layer have been subtracted (Fig A in S1 File).

After protein expression was initiated by the addition of plasmid DNA, two negative peaks immediately appeared at 1660 and 1550 cm^-1^ (Fig 3a). The negative bands were assigned to non-specifically bound protein of the expression system mix (Fig A in S1 File and Fig J in S6 File). Removal of the non-specifically bound supramolecular species, like large protein assemblies or carbohydrates prior to insertion of the peptide, is a reasonable scenario in an environment like the densely packed cytoplasm of a living cell. However, the cleansing process does not only cost free energy for membrane insertion but also slows down protein insertion. Although this scenario may seem trivial, it is the first *in-situ* observation of a clearing process prior to insertion of a polypeptide into a biomembrane.

The cleansing time of surface-bound macromolecules from the nanodiscs takes about 5 minutes (Fig 3a). After this, positive bands arise in the same spectral region, which are assigned to the nascent polypeptide chain inserting into the lipid bilayer.

### Membrane insertion of the polypeptide chain and formation of secondary structure (10–60 min)

At approximately 10 minutes after the transcription/translation was started, a weak but distinct positive amide I band of the nascent polypeptide backbone was assignable at 1645 cm^-1^ (Fig 3b) [34]. Slightly delayed, another amide I band appeared at 1679 cm^-1^. Both amide I bands got stronger as time proceeded, indicating progressive insertion of polypeptide into the lipid bilayer. The amide II band at 1557 cm^-1^ appeared after 18 minutes. As insertion proceeded over 50 minutes, the amide I signal at 1679 cm^-1^ shifted towards 1673 cm^-1^ while the component at 1645 cm^-1^ shifted to 1655 cm^-1^. Concomitantly, the amide II band at 1557 cm^-1^ shifted down to 1549 cm^-1^.

The frequencies of the amide vibrations are characteristic for the secondary structure of proteins. A steady increase in band intensity reflects continuous insertion of the polypeptide into the nanodiscs, while a band shift is indicative for secondary structure changes in the protein backbone. The frequency of the amide I band in the range of 1645–1650 cm^-1^ is characteristic for random structure (or partially solvated helices at the membrane surface) whereas the amide band above 1670 cm^-1^ is characteristic for the appearance of reverse turns or loops [35].

It is noted that the vibrational bands, which are assigned to characterize the folding process of bR, are observed only when a suitable biomimetic lipid membrane is present at the gold surface. This is shown by control experiments conducted in the absence of nanodiscs (Fig A in S1 File). Thus, we conclude that the observed bands are the result of folding related events rather than denatured and precipitated polypeptide. As a consequence of gradual changes of the secondary structure, the inserting polypeptides are converted into α-helical structures as evidenced by the shift of the amide I band to 1655 cm^-1^ [36].

In the time period between 15 and 60 minutes, the amide I peak shifts to 1655 cm^-1^ for which a rate constant of 0.0004 s^-1^ was derived for secondary structure formation assuming single exponential behavior. This value is about 15–20 times slower than secondary structure formation of bR as was reported from renaturation experiments of partially unfolded bR (0.006–0.008 s^-1^) [12, 14, 37]. The renaturation experiments start from a state comprising a substantial fraction of α-helical polypeptide [6, 7] whereas the nascent polypeptide chain generated during cell-free expression does not exhibit any secondary structure at early stages directly after synthesis as evidenced by the SEIRA spectra. Despite the fact that various factors can have influence on the observed differences, we infer that the rigidity of the membrane can seriously affect the folding. Booth et al. performed the refolding of bR into DMPC/DHPC micelles while the nanodiscs, which were used here, harbor DMPC, exclusively [38]. It has been suggested that the rate of helix formation is slowed down by increasing the relative proportion of DMPC in the mixed DMPC/DHPC micelles due to increasing rigidity to bending [38].

### Formation of tertiary structure (1–4 hrs)

Starting out from a relatively broad band with absorption maxima at 1655 and 1673 cm^-1^, the amide I band sharpened over time and the maximum is shifted to 1663 cm^-1^ (Fig 3c). The latter frequency is characteristic for polytopic α-helical proteins. The frequency up-shift of 6 cm^-1^ has been explained by vibrational coupling among helices when α-helical bundles form [39, 40].

Band narrowing is even more obvious when the amide I bands recorded at 63 minutes and 4 hours are compared (Fig 3c and Fig C in S2 File). It is shown that the component at 1655 and 1673 cm^-1^, assigned to single α-helices and turn structures, respectively, decreased over this time range and merged into a single band at 1663 cm^-1^. This result suggests that α-helices and turn structures are converted into a compact structure of a bundle of α-helices. Taken together, these observations lead us to conclude that the tertiary structure of the inserted polypeptide has been established during this time range and the folding process has completed. The synthesized and folded bR is fully functional as inferred from time-resolved UV/Vis spectroscopy performed on cell-free expressed bR into the nanodiscs in the batch experiment, which exhibited a photocyclic reaction including the rise of the M intermediate as a characteristic marker for proton translocation (See Fig K in S7 File). The kinetics of the photoreaction is identical to recently published experiments [41].

Along with peak narrowing, the absolute intensities of amide bands were also increasing. The increase was linked to the evolution of the tertiary structure along with increasing amounts of folded protein. The intensity increase was more pronounced for the amide I than for the amide II band in the time domain of the “membrane insertion and secondary structure formation (10–60 min.)”, while in the time domain of “tertiary structure formation (1–4 hrs.)” both rise equally (see Fig 2b). The stronger intensity of the amide I band can be explained by 1) an increase in packing of the transmembrane α-helices perpendicular to the membrane surface which leads to enhanced resonance of the transition dipole moments of the C = O stretches of the α-helices with the electromagnetic field along the plasmonic surface, (surface selection rule [31]) and 2) an increase in α-helical content upon tertiary structure formation which leads to vibrational coupling to provide a stronger transition dipole strength of the amide I mode [42]. As the dipole moment vectors of the amide I and II bands are orthogonal to each other, the increase of the absorption of the amide II band is less pronounced.

In conclusion, we interpret the relative intensity increase of the amide I versus the amide II band as an additional increase in α-helical content. The latter is explained by the model of Booth et al. [11, 15], where helices F and G of bR are formed only by the interaction with helices A to E. SMFS and AFM studies by Kessler et al. supported and refined this model [18, 43]. Thus, the first 60 minutes reflect insertion and folding of helices A to E, followed by insertion and folding of helices F and G accompanied by tertiary structure formation (see Fig D in S2 File).

### Effect of the prosthetic group on membrane protein folding

**Fig 4:**
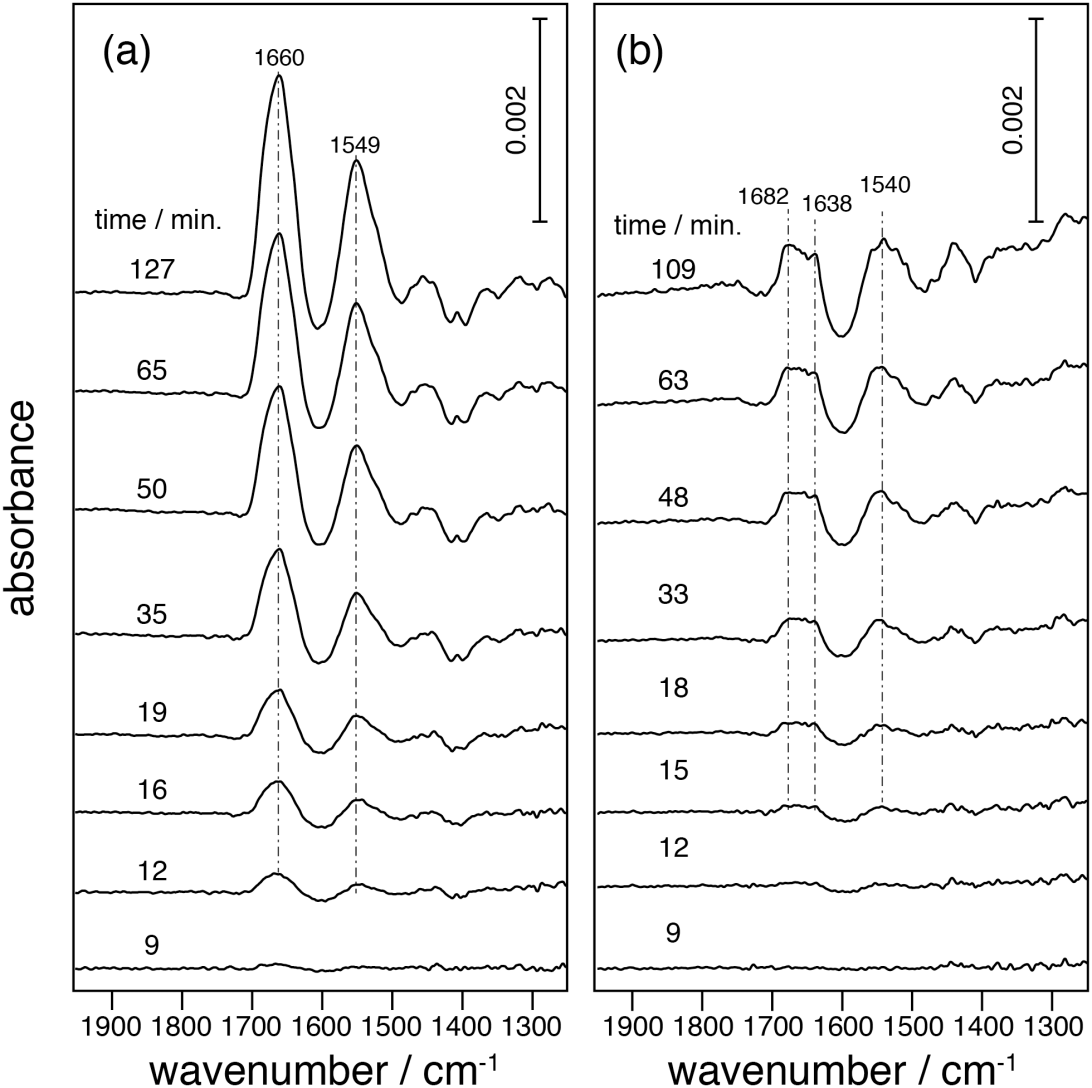
SEIRA spectra of insertion and folding of bO. (a) in the presence of retinal immersed in the nanodiscs, and (b) in the absence of retinal. Spectra in (b) are normalized by subtracting negative amide peaks raised from desorption of nonspecifically bound protein (see data in S3 File).

The influence of the chromophore all-*trans* retinal on folding of the polypeptide was determined by comparing experiments carried out in the presence (Figs 3 and 4a) or absence of retinal (Fig 4b). The data show that the presence of retinal significantly affects the folding process. In the absence of retinal only amide bands indicative for the presence of turns (loops) and irregular structures, at 1682 and 1638 cm^-1^, respectively, appear (Fig 4b), indicating that the synthesized polypeptide chain does not further assemble to secondary structure (see S4 File). Moreover, post-addition of retinal to the system does not result in recovery of the secondary structure (see S8 File). This is in contrast to the observations of the former refolding experiments, which suggests that functional bR can be fully recovered by post addition of retinal after denaturation [6, 7].

If the lipophilic retinal was immersed in the nanodiscs prior to starting protein expression, the obtained spectra showed amide I and II bands at 1660 and 1549 cm^-1^ (Fig 4a), which are characteristic frequencies for polytopic α-helical protein. This implies that freshly synthesized polypeptides fold into proper α-helical structures. The kinetics of α-helical formation is faster and tertiary structure is immediately established as indicated by the appearance of amide I bands at 1660 cm^-1^ (α-helical bundle) within the first 10 minutes. Secondary structure formation (single α-helix at 1655 cm^-1^) is not detectable because the succeeding tertiary structure forms rapidly under these conditions. If retinal was administered to the extract when starting the reaction, the folding process is much slower (Fig 3). It is evident that the nascent polypeptide interacts with pre-adsorbed retinal during membrane insertion, while in the experiment of Fig 3 slow diffusion of retinal from the bulk liquid solution phase to the hydrophobic lipid core retards helical formation. Evidently, a direct access to retinal during polypeptide insertion and folding is essential for proper helix formation to result in functional bR. The peak positions of the amide I band are slightly different (-3 cm^-1^) when bR was inserted in the nanodiscs with immersed retinal (Fig 4) and in nanodiscs with retinal in the extract (Fig 3). Both frequencies fall within the range characteristic to α-helical bundles but the frequency shift indicates slight differences in the final structure. To clarify this observation protein functionality in both conditions needs to be checked.

## Discussion

Membrane protein insertion and folding into a solid-supported lipid bilayer was studied by mid-IR spectroscopy during cell-free expression. The short-ranged enhancement of the electromagnetic field (about 10 nm) along a rough gold surface renders surface-enhanced vibrational spectroscopy an exquisite approach to selectively observe processes taking place in lipid bilayers (thickness of the surface-adhered nanodiscs is about 5 nm). The sensitivity of the amide I band (C = O stretching vibration) was used to analyze structural and orientational changes of the polypeptide during formation of primary, secondary and tertiary structures. Opposite to re-folding experiments from a (partially) denatured state, we observed folding from freshly synthesized bR under near-native conditions.

**Fig 5:**
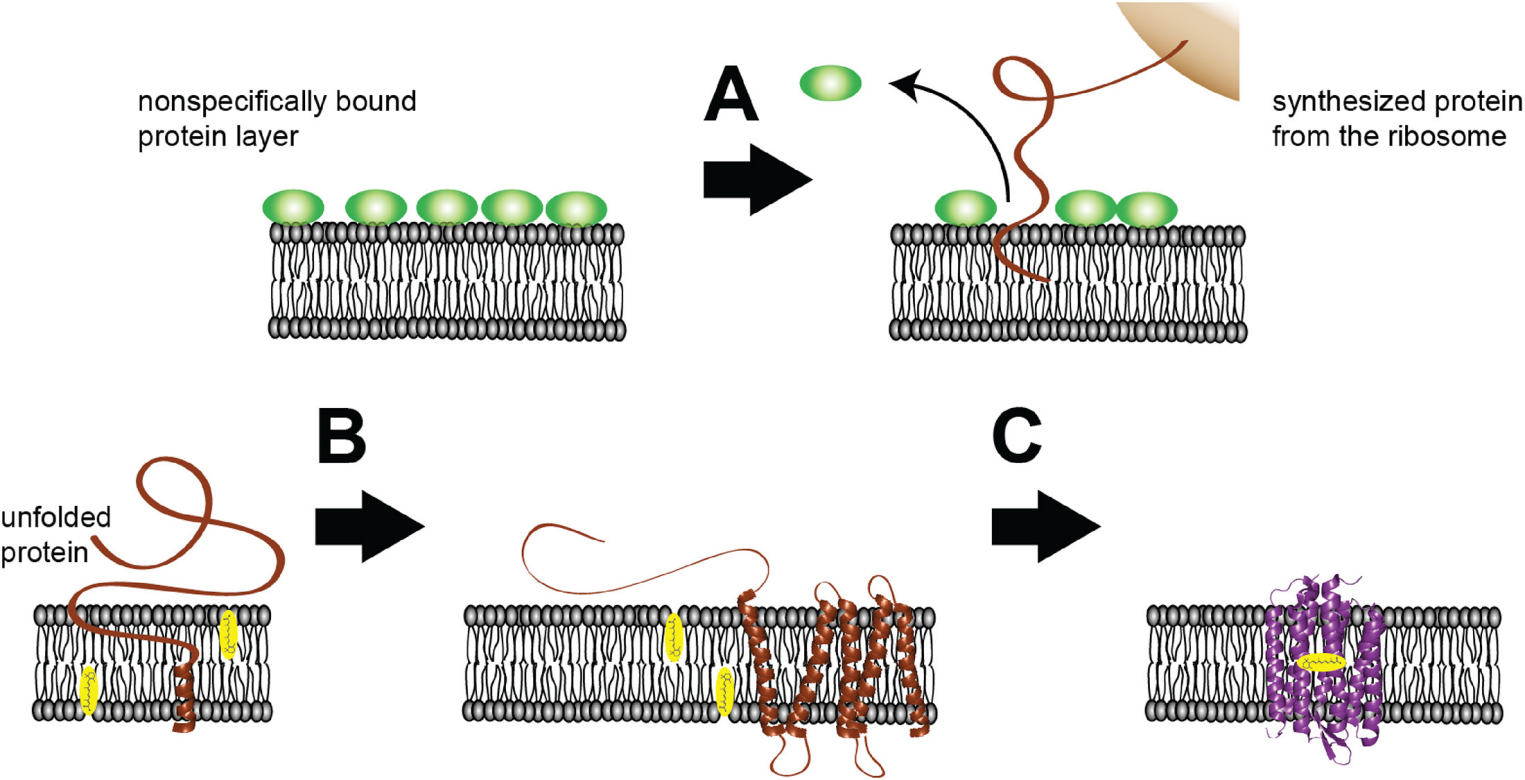
Models derived from SEIRAS observations. Before addition of the plasmid, the nanodisc monolayer is covered by nonspecifically bound protein (green spheres) of the *E*. *coli* cell extract. As DNA encoding bO is added, transcription and translation are initiated. Subsequently, the nascent polypeptide chain approaches the lipid bilayer surface (brown line). (A) Pre-adsorbed species are removed from the surface and the synthesized peptide inserts into the membrane. (B) During insertion, the peptide folds into individual transmembrane α-helices (brown helices). (C) Individual α-helices interact with each other, leading to folding of the final two helices G and F and formation of tertiary structure. Retinal (in yellow) at the lipid bilayer binds to the apo-protein and fully functional bR is formed. Tertiary structure formation is enhanced when retinal is pre-bound to the lipid bilayer.

The model derived from the vibrational analysis of the time-resolved SEIRA spectra is illustrated in Fig 5. We observed insertion and folding of the nascent polypeptide to proceed in separate and well-defined stages, which supports models proposed by various authors [1, 2, 11, 15, 43–45]. In the classical “two-stage model”, insertion of the polypeptide chain into the lipid bilayer takes place simultaneously with secondary structure formation (first stage). This reaction is followed by the association of the secondary structure elements leading to the functional tertiary structure (second stage). These models consider folding of α-helical membrane proteins not assisted by any chaperone or translocon. Deduced from the *in-vivo* results by Dale and Krebs [46], co-translational insertion is assumed for bR folding after cell-free expression. According to our results, insertion of the polypeptide chain into the lipid bilayer initially takes place followed by secondary structure formation. Yet, it is not clear where the ribosomes are located that support co-translational insertion. We assume that the ribosomes are adsorbed on the nanodiscs´ surface when the cell extract was added (prior to the addition of the plasmid DNA). In that case, IR absorption by the ribosomes is subtracted as background and would appear in the spectra taken during the translation process. The distance from the gold to the lipid bilayer surface is already at the limit of effective enhancement length of SEIRAS. Therefore, we do not expect substantial signals from the ribosomes even if they would approach the surface during translation and insertion. As soon as secondary structures are available, helix condensation starts and leads to functional tertiary structure.

The two-stage model is based on a series of experiments demonstrating that isolated TM fragments of bR in lipid bilayers are able to spontaneously re-assemble into a fully functional protein [15–17, 44]. This model sets a thermodynamic basis for ‘re-folding‘ of helical membrane proteins. Recent studies refine the model by proposing various sub-phases. Our results qualitatively fit to these models. We demonstrated here that the availability of the retinal cofactor is key to the folding process. This conclusion was drawn from the result that the presence of retinal immersed in the lipid phase, accelerates secondary and tertiary structure formation, while its absence leads to incomplete folding or even misfolding. Therefore, we conclude that retinal acts not only as a photon absorbing entity but is also crucial for effective structure formation at an early stage of protein folding. This is in line to the observations by Curnow and Booth that the presence of retinal stabilizes the protein during unfolding by increasing the rigidity and compactness of the polypeptide chain [47].

Although our observation of the folding events are qualitatively in line with previously reported results on unfolding and re-folding experiments, clear discrepancies are noted in the folding kinetics. As a typical example, Booth et al suggested that the rate-limiting step of folding to the native tertiary structure is the formation of α-helices [12]. These experiments started out from SDS-denatured bO, which containedα-helical structures equivalent to about four transmembrane helices. This α-helical intermediate can covalently bind retinal and transforms into functional bR. The latter process involving retinal binding and tertiary structure formation is supposed to be faster than the secondary structure formation from completely unfolded bR in acidic solution although the kinetics of this process is still under debate [12]. In contrast, the slowest process in our cell-free expression experiments is the formation of tertiary structure, which takes hours. Here, the folding rate of the membrane protein may be limited not only by the folding itself but also by other factors such as the rate of translation.

Since the physical and chemical conditions of our experiment are fundamentally different from those discussed, direct comparison of the results may not be appropriate. It must be pointed out, however, that our experimental approach offer the unique possibility to observe the folding process of a membrane protein while protein expression is running (*in operando*).

In conclusion, our results provide a proof-of-principle study of tracing insertion and folding of a membrane protein into a lipid bilayer with the inherent molecular sensitivity and temporal resolution of vibrational spectroscopy. The presented methodology integrates cell-free membrane protein expression into nanodiscs with SEIRAS and will provide sufficient flexibility to expand on the folding reaction of other membrane proteins, may it be spontaneous or translocon-assisted. It is non-invasive and offers immediate molecular insight into the pathways of protein folding during biosynthesis.

## Materials and Methods

### Cell-free protein expression

Cell-free protein expression based on *E*. *coli* was set up using MembraneMax^™^ HN Protein Expression Kit (Invitrogen) according to the manufacturer’s direction. The major components of the kit include: polyhistidine-tagged nanodiscs with DMPC (1,2-dimyristoyl-sn-glycero-3-phosphocholine) lipid bilayer, an optimized *E*. *coli* extract, reaction buffer composed of an ATP regeneration system, a feed buffer to replenish components, amino acids and T7 RNA polymerase. The used plasmid pEXP5-CT/bR with the gene coding for bO (bop), which is under control of the T7 promotor, is also part of the kit.

The buffer used in SEIRAS to generate the nanodisc monolayer consisted of 50 mM Na/PO_4_, pH 7.4 unless otherwise stated.

### In situ SEIRA spectroscopy

The experimental setup and procedures for SEIRAS have been described elsewhere [26,27]. Briefly, a thin gold film (thickness 100–200 nm) was formed on the reflection surface of a triangular silicon prism by chemical deposition. After electrochemical cleaning, the gold-coated prism was set into a Teflon cell. The cell was mounted on a self-made attenuated total reflection optics (single reflection, 60° incident angle) built in the FT-IR spectrometer (Bruker Vertex 70V). The reflected beam was recorded with a mercury cadmium telluride (MCT) detector. Typically 100 to 512 scans were co-added for each spectrum depending on the time resolution. All SEIRA measurements were handled in H_2_O aqueous solution rather than D_2_O.

### Preparation of the nanodisc monolayer

#### Preparation of Ni-NTA SAM

The Ni-NTA layer was prepared on the gold surface as previously described [32]. After rinsing the surface with water, 200 μl with approximately 1 μM nanodiscs dissolved in 50 mM Na/PO_4_ buffer (pH 7.4) was added on top of the Ni-NTA coated Au surface. The adsorption of the nanodiscs via the 6xHis-tag at the N-terminus of the membrane scaffold proteins was typically conducted overnight. The adsorption of the nanodiscs was monitored by SEIRAS and is described in Fig I in S5 File.

### Insertion and folding of bR into the nanodisc monolayer

#### (1) Pre-treatment before transcription/translation

After the formation of the nanodisc monolayer, the SEIRAS cell was filled with 200 μl buffer and 50 μl reaction mix. The reaction mix contained all components for the cell-free expression including all-*trans* retinal except the *bop* containing plasmid. Since the plasmid DNA was missing in the solution, synthesis of the membrane protein did not take place. However, as the components of the kit, especially the undefined *E*. *coli* cell extract, contain a mixture of proteins, which could nonspecifically adsorb onto the nanodisc surface, SEIRA spectra were recorded to determine background signals (shown in Fig J in S6 File). When the IR bands from nonspecific adsorption reached their maximum due to adsorption equilibrium (approximately 1 hour), we proceeded to express bR.

#### (2) Transcription/translation and folding of bR

The transcription and translation was initialized by 0.5 μg plasmid in 50 μl feeding mix solution that was added to the mixture in the optical cell. Sample spectra were taken in a time-resolved manner with consecutive time intervals of 10 s (X 15 spectra), 60 s (X 15 spectra) for the first 30 spectra. Afterwards, the spectra were measured with time intervals of 5 min and 10 min (X 15 spectra respectively) with spectral co-addition of 60 s. Presented data were from selected single experiments. Reproducibility of the data was confirmed by repeating experiments under same conditions. The time points shown in spectral figures are the starting points of the selected measurement.

### Effect of the prosthetic group on protein folding

To determine the influence of the cofactor retinal, different approaches have been investigated. In a first approach, expression of bO was carried out under the same conditions as described before, but in the absence of all-*trans* retinal in the reaction mixture (Fig 4b). Only when the expression process reached equilibrium as derived from the IR absorption, 0.5 μl 10 mM all-*trans* retinal was added.

In another approach, immobilized nanodiscs were initially incubated with 25 μM all-*trans* retinal in buffer (40 mM Tris/HCl, 100 mM NaCl buffer, pH 8) until saturation is indicated by no further changes in absorption. The excess of retinal was removed by thorough washing. Thereafter, the experiment was continued as described for the default experiment with no additional retinal (Fig 4a).

## Supporting Information

S1 File Subtraction of background signal of Ni-NTA layer from the observed spectra. (PDF)

S2 File Surface structural model interpreted from the observed spectra during secondary and tertiary structure formation according to the surface selection rule of SEIRAS. (PDF)

S3 File Normalization of the SEIRA spectra of bO expressed in the absence of all-trans retinal. (PDF)

S4 File Determination of fractions of the secondary structures from amide I band of bO expressed in the absence of all-trans retinal. (PDF)

S5 File Structure of the nanodisc monolayer on a gold surface. (PDF)

S6 File Nonspecific adsorption of undefined species on the nanodisc SAM layer before transcription/translation. (PDF)

S7 File Functionality of the cell-free expressed bacteriorhodopsin. (PDF)

S8 File Post-addition of retinal to bO folding into nanodiscs. (PDF)

## Abbreviations

SEIRAS: Surface Enhance Infrared Absorption Spectroscopy
TM: transmembrane
Ni-NTA: nickel chelating nitrilotriacetic acid
bR: Bacteriorhodopsin
bO: bacterioopsin
DMPC: 1,2-dimyristoyl-sn-glycero-3-phosphocholine
