# Supplementary material for: In-Situ Observation of Membrane Protein Folding during Cell-Free Expression
Source: PLoS One. 2016 Mar 15;11(3):e0151051. doi: 10.1371/journal.pone.0151051 (PMC4792443; doi:10.1371/journal.pone.0151051)
Supplement: S1 File — (PDF) [file pone.0151051.s001.pdf]

S1: Subtraction of background signal of Ni-NTA layer from the observed spectra

Fig A shows raw SEIRA spectra, showing the main experiment and a control experiment without nanodiscs. The Fig 3 shown in the main text is a subtraction of both curves. Both spectra were obtained after initialization of transcription/translation (time = 0 s) by adding the plasmid coding for bO.. The primary changes were positive peaks at 1564 cm^{-1} with a shoulder at 1590 cm^{-1} and 1417 cm^{-1} (Fig A(a)). Intensities of these peaks increased within ca. 30 seconds, decreased subsequently and vanished completely after 5 minutes. These bands are assigned to partial deprotonation of carboxylic groups (the asymmetric and symmetric COO^{-} stretching modes, respectively) of Ni-NTA molecules underneath the nanodisc layer (Fig B). The addition of the provided feeding mix solution with the plasmid may differ in pH from the buffer solution (pH 7.4) overlaying the Au surface, leading to a short pH jump visible in the spectra. As appearance of the bands from Ni-NTA layer hampered the analysis of protein, which appears in the same spectral regions we subtracted both spectra in the manuscript.

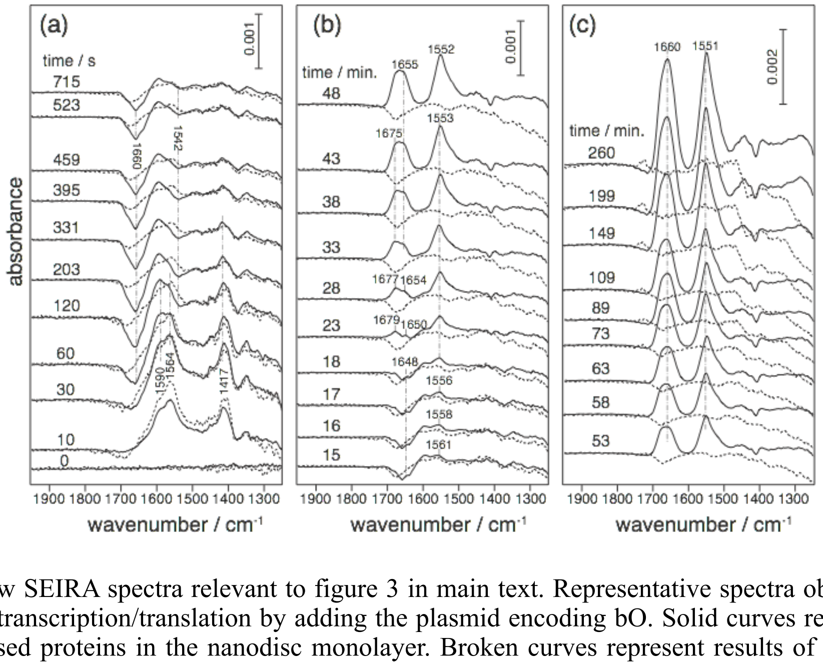

Figure A: A set of raw SEIRA spectra relevant to figure 3 in main text. Representative spectra obtained in each phase after initialization of transcription/translation by adding the plasmid encoding bO. Solid curves represent insertion and folding of the expressed proteins in the nanodisc monolayer. Broken curves represent results of a control experiment that has been done under the same conditions but without a nanodisc monolayer on the Au surface.

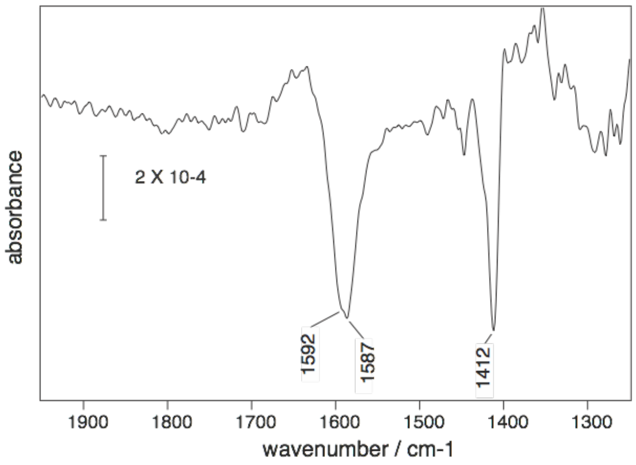

Figure B: pH induced SEIRA difference spectrum of Ni-NTA SAM layer. Reference spectrum was taken in 20 mM HEPES, 150 mM NaCl buffer with pH=8.5. 1/10 volume of 0.1 M HCl was added to the cell,which led to a final pH=7.5. This spectrum is only observed within the first 10 seconds after addition of HCl, when the local pH in the vicinity of the Au surface becomes momentarily lower.
