# Supplementary material for: In-Situ Observation of Membrane Protein Folding during Cell-Free Expression
Source: PLoS One. 2016 Mar 15;11(3):e0151051. doi: 10.1371/journal.pone.0151051 (PMC4792443; doi:10.1371/journal.pone.0151051)
Supplement: S2 File — (PDF) [file pone.0151051.s002.pdf]

S2: Surface structural model interpreted from the observed spectra during secondary and tertiary structure formation according to the surface selection rule of SEIRAS

Observations of the spectral change obtained after secondary and tertiary structure formation (time after 1hr ~ 4 hrs, see also Fig C) indicate an increase of amide intensities along with peak narrowing. A sequence of folding processes of bR is proposed in Fig D. The most probable interpretation for the increase of the amide band intensities is the net increase of α-helical dipole moment coupling with the enhanced electromagnetic field (see the red arrows representing the amide I dipole components of the α-helices in Fig D). In the period of secondary structure formation (15 min to 1 hour), the dipole component of each individual α-helix is in arbitrary direction, thus the coupling with the enhancing electromagnetic field of SEIRAS (blue arrows at right) is weak. In the period of the tertiary structure formation (1 to 4 hours), dipoles of each transmembrane α-helical component are coupling in the direction of the enhanced EM field.

According to Booth et al., the two C-terminal helices (F and G) only form α-helices when they are stabilized during tertiary structure formation by the interaction with the other helices (A to E). So an additional contribution of amide bands increase is due to this described effect (Fig D).

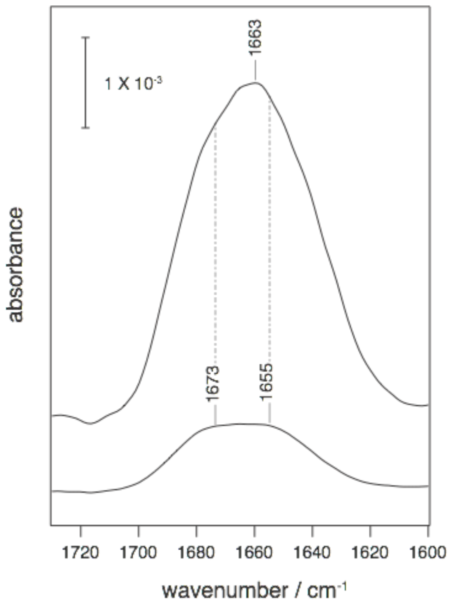

Figure C: Comparison of the spectral features of amide bands obtained at an early stage (lower curve, after 53 minutes) and at a late stage (upper curve, after 260 minutes, taken from Fig 3c). Relative decrease of bands at 1673 and 1655 cm^{-1} suggest that these secondary structure components were declining after 1hr, and the increase of 1663 cm^{-1} component suggest growth of α-helical bundles.

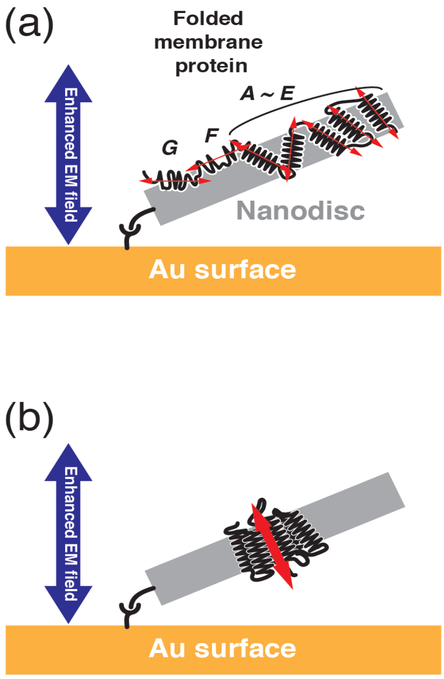

Figure D: Models interpreted from the observed spectra during (a) secondary and (b) tertiary structure formation according to the surface selection rule of SEIRAS. Narrow red arrows represent dipole components in the α-helices. During secondary structure formation, the dipole component of each individual α-helix is in arbitrary direction, thus the coupling with the enhanced electromagnetic field of SEIRAS (thick blue arrows) are small. During the tertiary structure formation, dipoles of each transmembrane α-helical component are coupled in one direction. Such directional dipole can strongly couple with the enhanced EM field.
