# Supplementary material for: In-Situ Observation of Membrane Protein Folding during Cell-Free Expression
Source: PLoS One. 2016 Mar 15;11(3):e0151051. doi: 10.1371/journal.pone.0151051 (PMC4792443; doi:10.1371/journal.pone.0151051)
Supplement: S3 File — (PDF) [file pone.0151051.s003.pdf]

S3: Normalization of the SEIRA spectra of bO expressed in the absence of all-trans retinal

Fig E presents spectra of membrane protein insertion/folding processes in the absence of retinal chromophore of (a) raw data and (b) after the subtraction procedure (the latter is shown in Fig 4 in the main text). In Figure E(a) contributions from inserting proteins, which give positive bands, overlap with desorption of nonspecifically bounds proteins contributing as negative bands. This condition hampers seriously the analysis of correct amide I band shape. From analysis of the peak height at 1661 cm^{-1} of the desorbing species, the negative band's contribution becomes maximal at 6.6 minutes. Thus we employed the spectrum at 6.6 minutes as a reference of all subtraction processes (for an example see Fig F).

The subtraction leads to a perfect straight line for spectra between 6.6 and 9 minutes (see baseline at 8.7 min in Fig E(b)). As insertion of the polypeptide does not occur at this time, it proofs that the background subtraction is correct. It should be noted that there is still a strong negative contribution at around 1600 to 1650 cm^{-1} after the subtraction. This negative peak is attributed to HOH bending mode of water molecules that expelled at the lipid surfaces as protein insertion proceed. This assignment is confirmed by the concomitant appearance of a strong negative peak at around 3400 cm^{-1}, which can be assigned to OH stretching mode.

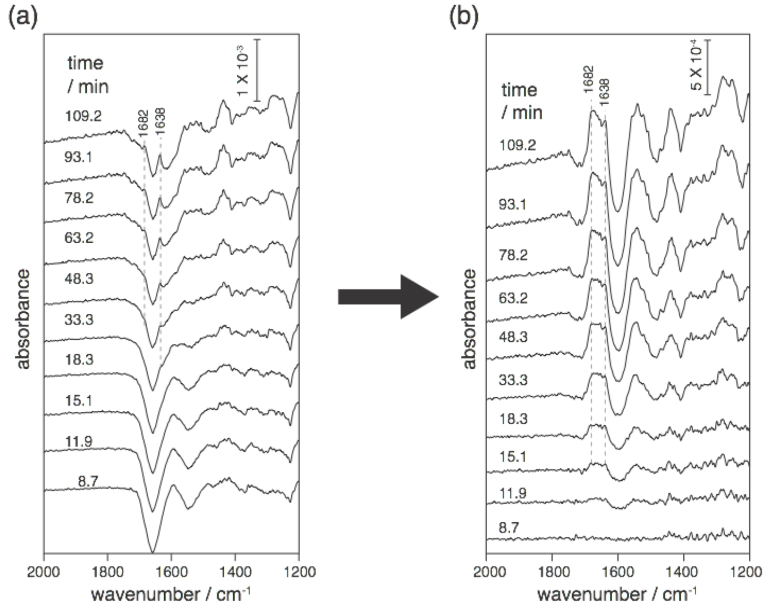

Figure E: The normalization of the SEIRA spectra obtained during transcription/translation of bO in absence of retinal. (a) Spectra observed before normalization and (b) after normalization. The normalized spectra were obtained by subtraction of a spectrum recorded at 6.6 minutes (395 seconds) after initialization of the expression. The subtraction factor was fixed at 1 for all subtraction processes.

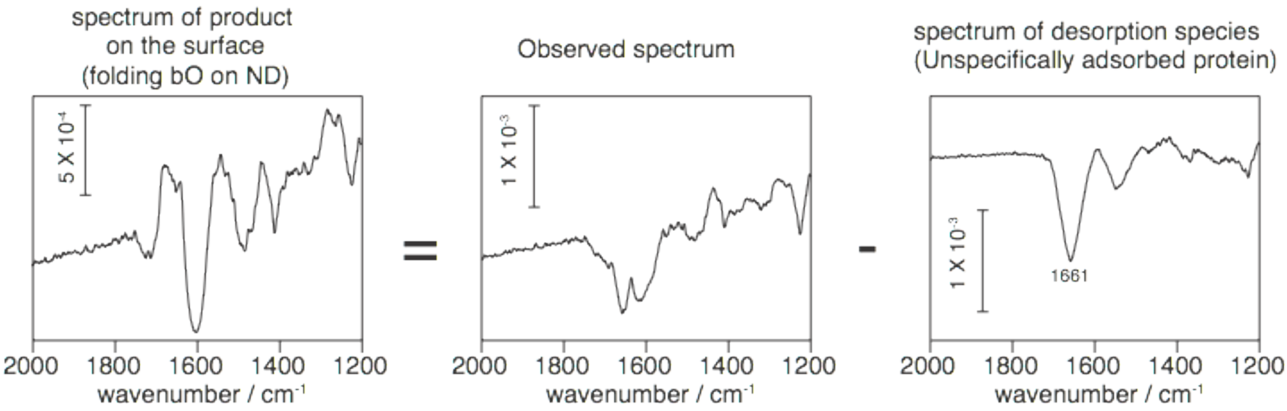

Figure F: An example of the normalizing process shown in Fig E. Observed spectrum (middle) contains contributions from desorption and insertion of proteins. At 6.6 minutes after transcription/translation, the contributions from desorption is maximal. Thus, the spectrum at 6.6 minutes is selected as reference (right). The resultant spectrum mainly represents the inserting protein with minor contributions of desorbing water molecules (left).
