# Supplementary material for: In-Situ Observation of Membrane Protein Folding during Cell-Free Expression
Source: PLoS One. 2016 Mar 15;11(3):e0151051. doi: 10.1371/journal.pone.0151051 (PMC4792443; doi:10.1371/journal.pone.0151051)
Supplement: S4 File — (PDF) [file pone.0151051.s004.pdf]

S4: Determination of fractions of the secondary structures from amide I band of bO expressed in the absence of all-trans retinal

As mentioned in the main text, absence of retinal leads to a completely misfolded structure that mostly consists of loops and irregular polypeptides. A post-addition of retinal molecules to the system after the transcription/translation reached an equilibrium (ca. 4 hours) did not help to recover secondary structure formation. This result suggests that retinal needs to be present in an early state of bO synthesis to obtain its fully folded functional (holo-) state bR. For the cell-free expressed bO without retinal, rough estimation of the α-helical content is determined by peak fittings of the amide I band (Fig G).

It is known that single peaks of various secondary structure components overlap in the amide I band. Although the fine structure can be determined by increasing fitting components, we restrict fitting to three components to simplify a determination of sole α-helical content. These three components consist of loops at >1670 cm^{-1}, α-helices at 1652-1665 cm^{-1}, and irregular structures at <1650 cm^{-1}. Fig G shows the results of the fitting of amide I band after 48 minutes in figure 4 of the main text. The dashed curve in Fig G(a) represents the shape of the original amide I band, while the thick solid curve represents the fitted band (thin broken curve represents the baseline). The three components are shown as solid curve in Figure G(b), respectively. The residual spectrum after subtraction of these three components, shown in the dashed curve in Fig G(b), reveals a straight line within the noise level. The peak intensities and other fitted parameters obtained for each component are tabulated in Supplementary table H.

The fraction of the α-helical component was calculated from the ratio of integrated peak area by following equation:

$$\% (\alpha\text{-helix}) = A_{(1659\ cm-1)} / (A_{(1638cm-1)}+A_{(1659cm-1)}+A_{(1682cm-1)})$$

This results suggest that the α-helical content is less than 38 % in the entire structure in the absence of retinal giving rise that the essential core structure failed to form .

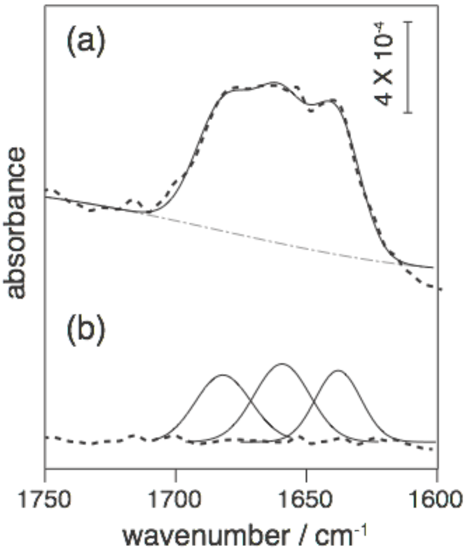

Figure G: Result of spectral peak fitting of observed amide I band at 48 minutes in Fig 4(b) of the main text. Fitted spectrum is represented as solid curve in (a), while broken curve represents original spectrum. Thin dashed curve is baseline, tilted due to a contribution of desorbing water. Broken curve in (b) presents residual spectrum after subtraction of fitted curve and baseline from original spectrum. Solid curve in (b) presents fitted peak components. Details of fitted components are shown in Table H.

	Type	peak position / cm^{-1}	assigned polypeptide structure	peak height / absorbance	integrated peak area / A(ν)	FWHM	secondary structure fraction / %
amide I	Gauss	1638	irregular	3.10 X 10^{-4}	6.75 X 10^{-3}	2.05 X 10^{1}	28.8
	Gauss	1659	α-helix	3.39 X 10^{-4}	9.00 X 10^{-3}	2.50 X 10^{1}	38.3
	Gauss	1682	loop	2.90 X 10^{-4}	7.72 X 10^{-3}	2.50 X 10^{1}	32.9

Table H: Results of peak fitting of amide I band shown in Figure G. For each peak component, a Gaussian type peak has been applied.
