# Supplementary material for: In-Situ Observation of Membrane Protein Folding during Cell-Free Expression
Source: PLoS One. 2016 Mar 15;11(3):e0151051. doi: 10.1371/journal.pone.0151051 (PMC4792443; doi:10.1371/journal.pone.0151051)
Supplement: S5 File — (PDF) [file pone.0151051.s005.pdf]

S5: Structure of the nanodisc monolayer on a gold surface

A SEIRA spectrum of the nanodisc monolayer is shown in Fig I. A reference spectrum has been taken from the Ni-NTA SAM modified Au surface in a buffer solution (200 µl, Na/PO4, pH 7.4). After addition of 200 µl of ca. 1 µM nanodisc to the buffer solution, the sample spectra were recorded sequentially. The spectrum shown in Fig I was taken after 100 minutes of incubation. The spectra did not change further in their intensities and features after 100 minutes, implying that the nanodisc monolayer reached its adsorption equilibrium. The observed spectrum implies bands from the Ni-NTA layer and the nanodiscs adsorbed on the Au surface. The bands, which appeared between 1800 and 1500 cm^{-1}, were assigned to vibrations of the surface confined nanodiscs.

Two prominent bands, which appear at 1655 and 1551 cm^{-1}, were assigned to amide I and II, respectively, of the scaffold protein. A small band at 1738 cm^{-1} was assigned to C=O stretching mode of an ester group at the polar head of the DMPC lipid bilayer.

It should be noted that the relative intensity of the lipid band (1738 cm^{-1}) was small compared to the amide bands of the scaffold protein although one nanodisc contained approximately 160 lipids per disc (80 per leaflet) while two apo-lipoproteins are part of one nanodisc. This could be explained in terms of the orientation of the nandiscs and the surface selection rule of SEIRAS. Within the lipid bilayer, the dipoles of the C=O groups of the lipids polar head groups were assumed to be oriented parallel to the disc surface. Since SEIRAS has the property that bands from dipoles oscillating parallel to the gold surface appear weak in the spectrum (surface selection rule), the signal from C=O groups would be significantly reduced when the orientation of the nanodisc surface is almost parallel to the subjacent gold surface. Zaitseva et al. roughly estimate the angle between the surface tethered nanodisc and the gold substrate to be 24° (± 10°) from the ratio of $\nu_{as}(CH_2)$ and $\nu_s(CH_2)$ at around 2900 cm^{-1}. We conclude a slightly higher angle of the nanodiscs in our system, due to the higher amide I/II ratio of our SEIRA spectra compared to the reported one.

Nevertheless, tilted adsorption of the nanodiscs with respect to the gold surface could reduce the relative intensity of the C=O band as it was observed. The bands below 1500 cm^{-1} are observed when any kind of protein is adsorbed via His-tag to Ni-NTA. Therefore, we assigned these bands to be raised by Ni-NTA and His-tag interaction. Although detailed analysis of these bands are beyond the scope of this study, we tentatively assigned the bands at 1433 and 1364 cm^{-1} to the $\nu_s(COO^-)$ mode of the carboxylate group of NTA ligated to a Ni^-ion (Fig I).

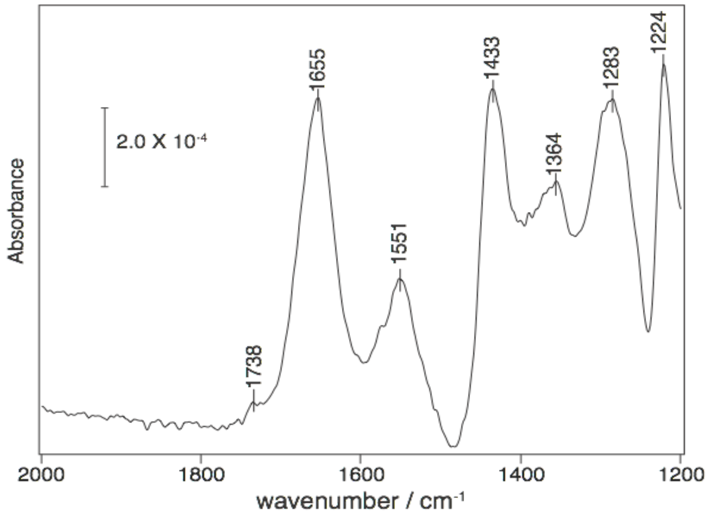

Figure I: the SEIRA spectrum of the nanodisc monolayer bound to a Ni-NTA SAM modified Au film via a His-tag.
