# Supplementary material for: In-Situ Observation of Membrane Protein Folding during Cell-Free Expression
Source: PLoS One. 2016 Mar 15;11(3):e0151051. doi: 10.1371/journal.pone.0151051 (PMC4792443; doi:10.1371/journal.pone.0151051)
Supplement: S6 File — (PDF) [file pone.0151051.s006.pdf]

S6: Nonspecific adsorption of undefined species on the nanodisc SAM layer before transcription/translation

Fig J presents spectra of species nonspecifically bound to the nanodisc monolayer after addition of the master mixture from *E. coli* cell extract. We observed immediate increase of bands at 1659 and 1548 cm^{-1}. As long as DNA was absent the transcriptional / translational process did not take place at this point. The peak positions and shapes of these bands resemble signals from amide I and II, so the nonspecifically adsorbing species are most likely proteins from the *E. coli* cell extract, which remain unidentified. As the characteristic bands from His-tag/NiNTA interaction below 1500 cm^{-1} (see Fig I in S5 file) are missing, this protein does not bind to Ni-NTA layer, but rather binds to the lipid surface of the nanodisc layer.

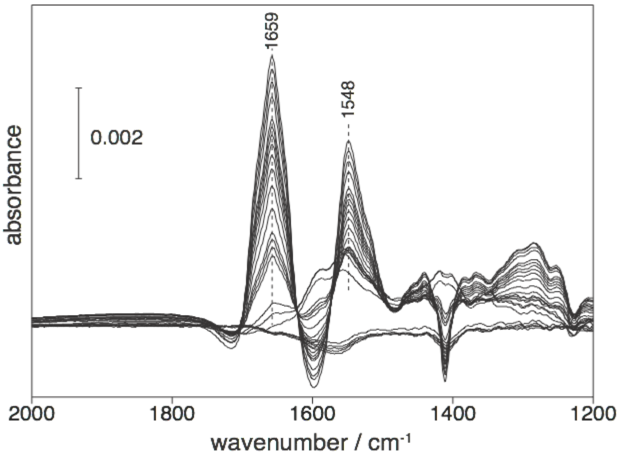

Figure J: SEIRA spectra obtained from the nanodisc monolayer after addition of the cell-free expression components without the bO encoding plasmid in variable times. Increase of 1659 and 1548 cm-1 (assigned to amide I and II, respectively) suggests adsorption of protein like species on the nanodiscs.
