# Supplementary material for: In-Situ Observation of Membrane Protein Folding during Cell-Free Expression
Source: PLoS One. 2016 Mar 15;11(3):e0151051. doi: 10.1371/journal.pone.0151051 (PMC4792443; doi:10.1371/journal.pone.0151051)
Supplement: S7 File — (PDF) [file pone.0151051.s007.pdf]

S7: Functionality of the cell-free expressed bacteriorhodopsin

The functionality of the cell-free expressed bR in nanodiscs has been analyzed. The photocycle can be measured by laser flash photolysis at representative wavelengths. The sample was excited by a short pulse from a solid-state laser (Nd:YAG, 10 ns laser pulse at 532 nm, energy density of 3 mJ/ cm^2, one pulse every 10 seconds). The flash photolysis experiments in the UV/Vis range were performed on two time scales, with the faster time range (<300 μs) recorded with the light source (Xe arc lamp) in pulsed mode and the slower time scale (>30 μs) recorded with the lamp in continuous operation. Data were averaged on a quasi-logarithmic time-scale and merged to yield time traces covering the time range from 500 ns to 10 s. Ten kinetic traces were averaged at each selected wavelength. The absorbance kinetics at 410 (red), 570 (blue), and 650 (green) nm in Fig K indicate the fraction of the M intermediate, ground state and O intermediate, respectively. They show typical kinetics of bR photocycle reconstituted in DMPC lipid bilayer, and demonstrate that the synthesized bR in the nanodiscs is fully functional. Based on this observation, we suppose that the bR in the surface-tethered nanodiscs reveal the same activity and functionality as in the batch experiment.

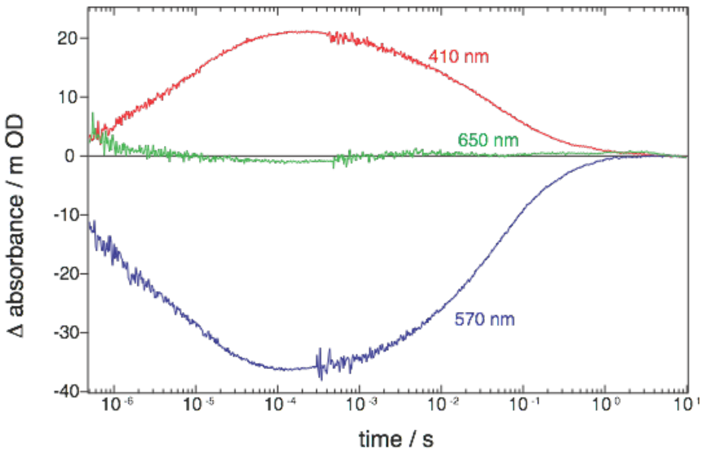

Figure K: Flash-photolysis kinetics of bR in nanodiscs obtained from cell-free expression of bR into nanodiscs in a reaction tube under comparable conditions as done on the gold surface in a SEIRAS experiment.
