# Supplementary material for: In-Situ Observation of Membrane Protein Folding during Cell-Free Expression
Source: PLoS One. 2016 Mar 15;11(3):e0151051. doi: 10.1371/journal.pone.0151051 (PMC4792443; doi:10.1371/journal.pone.0151051)
Supplement: S8 File — (PDF) [file pone.0151051.s008.pdf]

S8: Post-addition of retinal to bO folding into nanodiscs

Fig L shows a series of IR spectra taken after late addition of the retinal to already expressed bO in nanodiscs. Apo-protein, bacterioopsin, was expressed in the cell-free system without addition of retinal for 4.5 hours (See figure 4b in the main text). A new background spectrum was taken at this time point. In order to see an effect on folding post-translationally by addition of retinal, 17 μM retinal was added to the solution and the sample spectra were taken. In Fig L solely changes affected by the addition of the retinal are observed. IR absorption bands appeared at 1735, 1688, 1566, 1535 cm^{-1}, which were assigned to retinal molecules adsorbed to the nanodiscs. Despite the appearance of these retinal bands, no changes of the amide mode from the apo-protein could be observed. This result suggests that the post-addition of retinal did not induce correct folding of the bO.

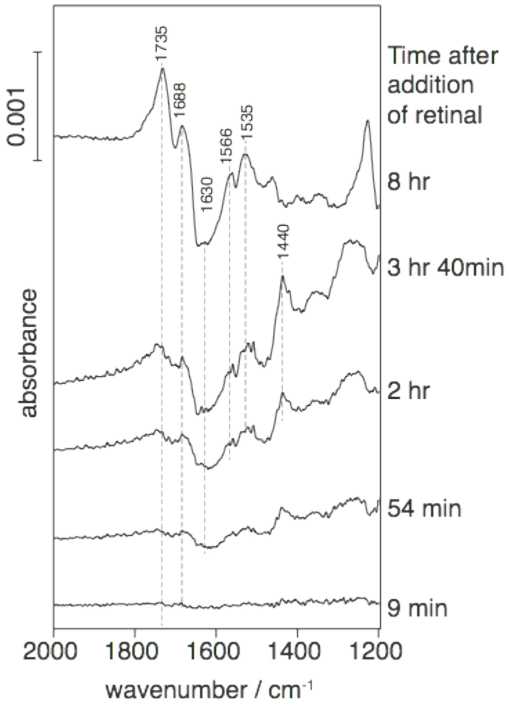

Fig L: SEIRA spectra after addition of retinal to misfolded bO in nanodiscs. A background spectrum was taken after bO was cell-free expressed for 4.5 hours. Then, 17 μM retinal was added to the solution. Spectra were taken after times as indicated.
